# Effects of trypsinization and of a combined trypsin, collagenase, and DNase digestion on liberation and *in vitro* function of satellite cells isolated from juvenile porcine muscles

**DOI:** 10.1007/s11626-018-0263-5

**Published:** 2018-05-21

**Authors:** Claudia Miersch, Katja Stange, Monika Röntgen

**Affiliations:** Leibniz Institute for Farm Animal Biology (FBN), Institute of Muscle Biology and Growth, Growth and Development Unit, Wilhelm-Stahl-Allee 2, 18196 Dummerstorf, Germany

**Keywords:** Pig, Muscle tissue, Trypsin, Collagenase, Myogenic differentiation

## Abstract

Muscle stem cells, termed satellite cells (SC), and SC-derived myogenic progenitor cells (MPC) are involved in postnatal muscle growth, regeneration, and muscle adaptability. They can be released from their natural environment by mechanical disruption and tissue digestion. The literature contains several isolation protocols for porcine SC/MPC including various digestion procedures, but comparative studies are missing. In this report, classic trypsinization and a more complex trypsin, collagenase, and DNase (TCD) digestion were performed with skeletal muscle tissue from 4- to 5-d-old piglets. The two digestion procedures were compared regarding cell yield, viability, myogenic purity, and *in vitro* cell function. The TCD digestion tended to result in higher cell yields than digestion with solely trypsin (statistical trend *p* = 0.096), whereas cell size and viability did not differ. Isolated myogenic cells from both digestion procedures showed comparable proliferation rates, expressed the myogenic marker Desmin, and initiated myogenic differentiation *in vitro* at similar levels. Thus, TCD digestion tended to liberate slightly more cells without changes in the tested *in vitro* properties of the isolated cells. Both procedures are adequate for the isolation of SC/MPC from juvenile porcine muscles but the developmental state of the animal should always be considered.

The skeletal muscle is a complex tissue composed of multinucleated muscle fibers surrounded by blood vessels, nerves, fat, and connective tissue. Although cells in muscle fibers are post-mitotic, the muscle retains its ability to grow, to maintain itself, and to regenerate damaged tissue by resident mononuclear stem cells, the so-called satellite cells (SC) (Yablonka-Reuveni 2011). SC are located in niches beneath the sarcolemma and the basal lamina (Mauro 1961). SC and their myogenic progenitor cells (MPC) can be released from muscle tissue by mechanical disruption and enzymatic digestion (Danoviz and Yablonka-Reuveni 2012). The isolation and *in vitro* analysis of SC help to unravel their exact function during myogenesis and to define their impact on myogenic processes such as postnatal muscle growth, muscle regeneration, and plasticity. During tissue digestion, enzymes dissociate cell-cell and cell-matrix contacts and break down the structure of muscle and connective tissue to release mononuclear cells. Successful cell dissociation depends on the type of tissue, the species, the age of the animal, the dissociation medium, the enzymes used, the temperature, and the incubation time (Santangelo 2008). Many enzymes are available for use in tissue dissociation, e.g. trypsin, pronase, dispase, collagenases, and various combinations of them (see Table 1). Trypsin is a serine protease produced and secreted as inactive trypsinogen in the pancreas. It has a high specificity for cleaving peptide bonds at the carboxyl side of the basic amino acids arginine and lysine (Santangelo 2008). Pronase is a mixture of non-specific proteases from *Streptomyces griseus* and digests proteins to free amino acids (Narahashi et al. 1968). However, both enzymes can damage the cell membrane and surface antigens of SC, leading to problems in SC viability and antigen-based cell sorting (Danoviz and Yablonka-Reuveni 2012). As an alternative that maintains membrane integrity, dispase, a gentle bacterial endopeptidase produced by *Bacillus polymyxa*, can be used (Stenn et al. 1989). Since tissue digestion usually involves destroying extracellular structures, collagenase alone or in combination with other enzymes (e.g., trypsin, pronase, dispase) is widely applied. Various collagenase subtypes exist, and they also contain several proteolytic side activities such as caseinase, clostripain, or trypsin. Collagenases target the peptide bonds in collagen, the main component of the muscle extracellular matrix (Mandl et al. 1953; Mandl et al. 1958).

**Table 1:** . Review of various porcine muscle tissue digestion procedures from literature

Tissue digestion with:	Dissociation medium	Concentration (*C*), temperature (*T*), incubation time (IT)	Muscle	Age of the species	SC enrichment after dissociation	References
Pronase	PBS, EBSS, medium	*C* 0.5–1.4 mg/ml, *T* 37°C, IT 40–90 min	SM, ST, BF, LD, PM, VM	Neonatal–adult	Pre-plating, Percoll gradients or none	(Alexander et al. 2010, Alexander et al. 2012, Baquero-Perez et al. 2012, Blanton et al. 1999, Doumit and Merkel 1992, Gao et al. 2017, Hathaway et al. 1991, Holzer et al. 2005, Mesires and Doumit 2002, Wilschut et al. 2008, Yi et al. 2001, Zhu et al. 2013)
Pronase followed by collagenase	PBS with 1% HEPES (pronase), DMEM-HG with 5% FCS (collagenase)	*C* 1–1.5 mg/ml pronase, 1.5 mg/ml collagenase XI, *T* 37°C, IT 60 min each	ST	Neonatal	Frequent pre-plating	(Wilschut et al. 2010a, Wilschut et al. 2010b)
Trypsin	Ca^2+^ and Mg^2+^- free EBSS, PBS	*C* 0.2–0.25% trypsin, *T* 37°C, IT 60 min	Hind limb muscle, SM, ST, LD	Fetus, neonatal	Pre-plating, Percoll gradients	(Hembree et al. 1991, Mau et al. 2008a, Mau et al. 2008b, Miersch et al. 2017, Will et al. 2012)
Collagenase	PBS with CaCl_2_, medium	*C* 0.5 mg/ml collagenase IV or 0.2% collagenase II, *T* 37°C, IT 60–120 min	SM, LD, ST, diaphragm	Neonatal–juvenile	Pre-plating	(Gao et al. 2017, Redshaw and Loughna 2012, Redshaw et al. 2010, Wang et al. 2016, Wang et al. 2012)
Collagenase with trypsin and DNase	PBS with 1% glucose (Ca^2+^ free)	*C* 1.5–1.9 mg/ml collagenase II, 0.25–0.31% (trypsin), 0.1–0.01% (DNase I), *T* 37°C, IT 3 × 20 min	SM, LD	Fetus, neonatal	20% Percoll gradient	(Nissen and Oksbjerg 2009, Nissen et al. 2005, Ortenblad et al. 2003, Perruchot et al. 2012, Theil et al. 2006)
Collagenase with dispase	PBS + 2.5 mM CaCl_2_, DMEM	*C* 0.2–1% collagenase B or D, 1.1–2.4 U/ml dispase II, *T* 37°C, IT 24–90 min	ST, SM	Newborn, juvenile	Frequent pre-plating	(Ding et al. 2017, Wilschut et al. 2010a)

*SM* Musculus (M.) semimembranosus, *ST* M. semitendinosus, *BF* M. biceps femoris, *PM* M. psoas major, *LD* Musculus longissimus dorsi, *VM* M. vastus medialis

Porcine skeletal muscle tissue digestion and SC isolation and cultivation were first described by Doumit and Merkel (1992). Subsequently, several similar or modified procedures have appeared in the literature with sometimes comprehensive differences in the used digestion procedures (Table 1). The published protocols were adopted originally from other species (e.g. rodents, ovine, human) (Dodson et al. 1986; Harper et al. 1987; Hathaway et al. 1991 Baroffio et al. 1993) and showed variations in the enzymes employed (types, concentration, combinations), dissociation medium, age of the animal, and muscles. Criteria regarding the choice of digestion protocol made by the authors are often not mentioned in the articles, and controlled studies comparing the various enzymes used for tissue dissociation are difficult to find. Thus, the aim of the present work was to compare a combined enzyme digestion procedure (trypsin, collagenase, and DNase, termed TCD), as developed by Ortenblad (2003), with a simple trypsin digestion regarding cell yield, viability, myogenic purity, and *in vitro* cell function.

Muscle tissues for SC isolation were obtained from early postnatal German Landrace piglets (4 to 5 d of age) that had a normal birth weight (1.34 ± 0.13 kg) and that were kept in the experimental pig unit of the Leibniz Institute of Farm Animal Biology, Dummerstorf, Germany. Animal husbandry and slaughter followed the guidelines set by the Animal Care Committee of the State Mecklenburg-Western Pomerania, Germany, based on the German Law of Animal Protection. The right and left *Musculus longissimus dorsi* (LD) and *Musculus semimembranosus* (SM) were removed as a whole, trimmed of visible connective tissue, and weighed. Dissected muscle tissue was washed and minced intensively with scissors before fractional enzymatic digestion was performed in a water bath with stirring at 37°C for 60 min (0.25–0.5 *w*/*v*%). After 30 min, the first fraction of dissociated cells was obtained by collecting the cell slurry supernatant; the further digestion process was stopped by adding growth medium (αMEM Eagle, 20% FBS, 100 U/ml penicillin/streptomycin, 2.5 μg/ml amphotericin B, and 0.05 mg/ml gentamycin [all PAN Biotech, Aidenbach, Germany]). To the remaining muscle fragments, fresh enzyme solution was added and they were digested for a second 30-min period. Thereafter, muscle digestion was stopped completely with growth medium and both cell fractions were pooled. Tissue digestion was either performed with 1× trypsin solution (0.25%, 4000 U/ml, Sigma Aldrich, Hamburg,Germany) or a combination of 1× trypsin solution with collagenase CLS I (0.2%, 285 U/mg, Biochrom, Berlin, Germany) and DNase I (0.01%, 4636.4 U/mg, AppliChem, Darmstadt, Germany). The pooled cell suspensions were vigorously triturated through a 25-ml pipette, washed, and filtered through gauze and fine nylon mesh (20 μm). Muscle-dissociated cells were subjected to Percoll (Sigma Aldrich) density gradient centrifugation (1800×*g* for 1 h) to enrich myogenic cells (Miersch et al. 2017 Mau et al. 2008b). The Percoll gradient contained layers of 70, 40, and 25%, and myogenic cells were collected from the 40/70% interface. Cell number, cell size, and viability were quantified by using the Countess Automated Cell Counter (Thermo Fisher Scientific, Darmstadt, Germany), which combines an image analysis algorithm with trypan blue staining for analysis. Cells were seeded in dishes coated with collagen type I (Greiner Bio-one, Kremsmünster, Austria) and cultured in growth medium in an atmosphere of humidified air—5% CO_2_ at 37°C. Medium was changed 24 h after seeding to remove unattached cells.

The percentage of Desmin-expressing cells was determined by microscopy and flow cytometry as published in Miersch et al. (2017). Briefly, cells were fixed in ice-cold methanol, blocked in PBS containing 10% rabbit serum (only for immunocytochemistry), and incubated overnight with mouse anti-Desmin antibody (clone D-33, DAKO, Hamburg, Germany, 1:80). After being washed, samples were incubated with a rabbit anti-mouse Alexa488 antibody (Thermo Fisher Scientific, 1:1000) for 1 h at room temperature. For immunocytochemistry, cell nuclei were additionally stained with DAPI (15 min, 1 μg/ml). Proliferation rates and viability were calculated by determining cell number changes between passages and trypan blue exclusion, respectively. For differentiation assays, cells were seeded on Matrigel-coated (growth factor reduced; 1:50 coating with αMEM, BD Biosciences, Heidelberg, Germany) plates and first cultivated in growth medium. Differentiation was initiated when cells were nearly 80% confluent, by switching to serum-reduced differentiation medium (αMEM Eagle with 2% FCS) supplemented with 50% conditioned differentiation medium. In order to obtain conditioned differentiation medium, differentiation assays were performed with freshly isolated porcine SC/MPC for 3 d before the supernatants were collected and centrifuged. At the first sign of fusions (after 5 to 6 d), cells were fixed with 4% paraformaldehyde, permeabilized with Triton X-100, blocked with rabbit serum, and incubated with the primary antibody, namely, anti-Desmin (DAKO, 1:80) or mouse anti-skeletal fast Myosin (MHC, clone MY-32, Sigma Aldrich, 1:400), overnight. After being washed with PBS, samples were incubated with a rabbit anti-mouse Alexa488 antibody (Thermo Fisher Scientific) for 1 h at room temperature, and subsequently, cell nuclei were stained with DAPI. For quantification of differentiation MHC + myotubes (≥ 2 nuclei) were encircled with Image J (1.49v) to determine myotube area.

Cell isolation was mainly performed as described by Mau et al. (2008b) and Miersch et al. (2017) who used tissue digestion with 0.25% trypsin and Percoll gradient centrifugation to enrich SC/MPC cells. In order to test whether a combined enzyme tissue digestion would increase cell yield or alter myogenic purity and/or *in vitro* cell function, cells were digested with trypsin (0.25%) in combination with collagenase type I (0.2%) and DNase (0.01%) and compared with trypsin digestion alone. The use of a combined enzyme digestion tended to result in a numerically higher cell yield per gram muscle (trypsin 16.6 × 10^5^ ± 5.3 × 10^5^ cells/g muscle, TCD 25.2 × 10^5^ ± 10.8 × 10^5^ cells/g muscle; paired *t* test *p* = 0.096, Fig. 1*a*) but an increased variability of this parameter was detected. Cell viability and cell size were not affected by the digestion procedure (Fig. 1*a*). To determine the relative myogenic purity of the cultures, the proportion of cells positive for Desmin (Desmin+) was investigated via immunofluorescence staining and flow cytometry during culture. Desmin is a muscle-specific intermediate filament protein that has been used in many studies to determine the proportion of myoblasts in cultures of porcine muscle-derived cells (Mau et al. 2008b Baquero-Perez et al. 2012). However, in our previous study, we showed that cells isolated by trypsin digestion express additional myogenic markers, e.g., Pax7 or Myogenin (Miersch et al. 2017). Neither a visual nor a quantitative difference was noticed in the proportion of Desmin+ cells between cultures prepared by trypsinization or TDC digestion procedure (Fig. 1*b*, *c*). After 6–8 d in culture, the percentage of Desmin+ cells was about 60% and peaked to over 80% at day 11 (Fig. 1*b*). To investigate the effect of TCD digestion procedure on *in vitro* cell function further, proliferation and differentiation capacity were assessed. Although total cell yield per gram muscle was significantly higher in the TCD cell isolates after 6–8 d in culture (trypsin 16.2 × 10^5^ ± 12.9 × 10^5^ cells/g muscle, TCD 30.8 × 10^5^ ± 21.4 × 10^5^ cells/g muscle; paired *t* test *p* = 0.037), probably because of the higher cell number directly after isolation, the proliferation rate was similar (trypsin 1.0 ± 0.51, TCD 1.2 ± 0.59; paired *t* test *p* = 0.441). Cell viability showed also no differences at this timepoint (trypsin 95.2 ± 1.9%, TCD 94.0 ± 3.9%, paired *t* test *p* = 0.445). As can be seen by immunostaining for Desmin and MHC (Fig. 2), cells from both digestion procedures showed a similar ability to differentiate into the myogenic lineage as they started to express MHC and to fuse to elongated multinucleated myotubes. Quantitative analysis of differentiation confirmed no measurable differences in the average myofiber area between trypsin and TCD-liberated cells (trypsin 2674 ± 724 μm^2^, TCD 2760 ± 676 μm^2^, paired *t* test *p* = 0.926).

**Figure 1:**
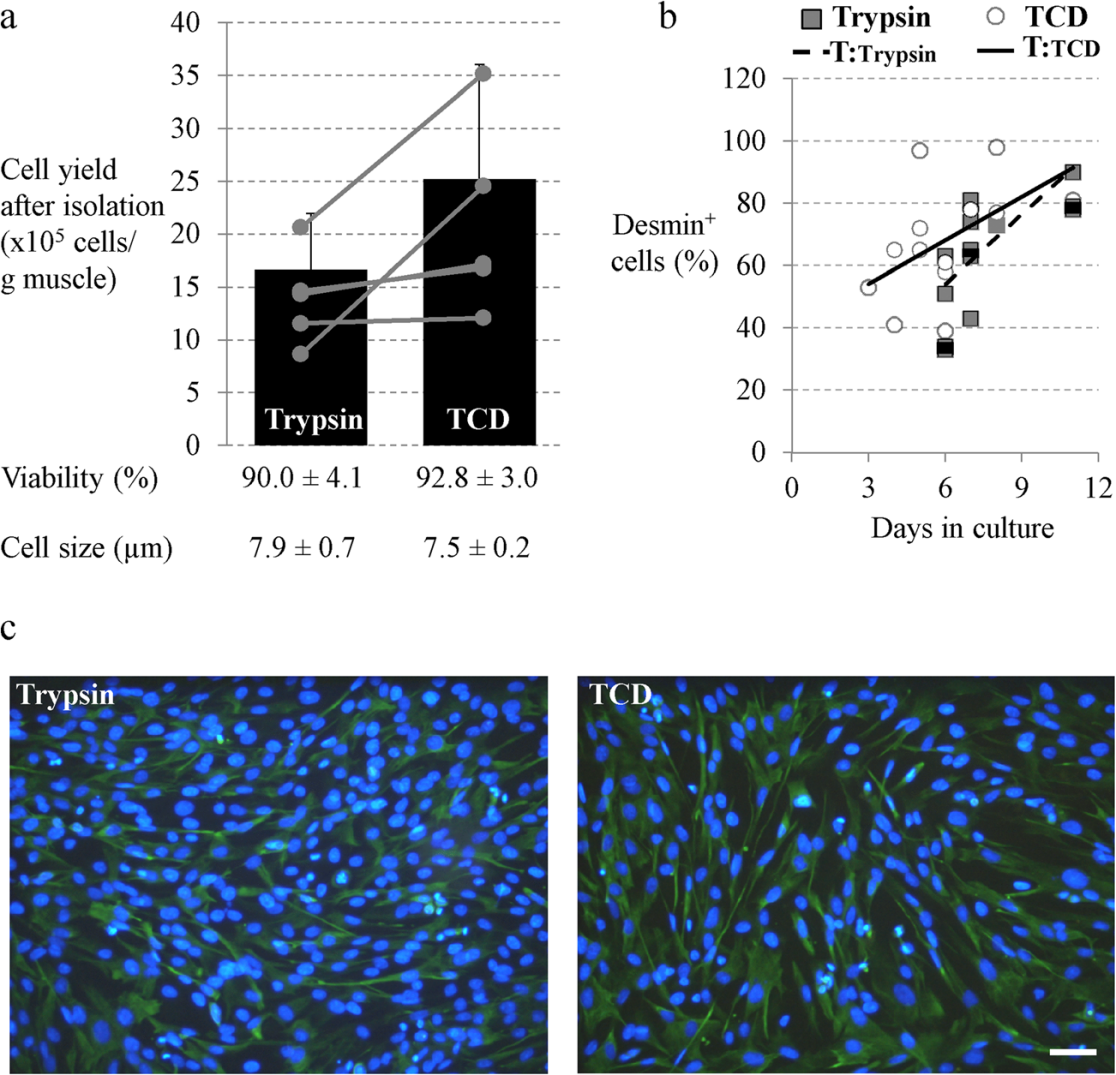
Cell characteristics and Desmin expression of satellite cells (SC)/myogenic precursor cells (MPC) liberated with trypsin alone or with a combined trypsin, collagenase, and DNase digestion (TCD). *a*–*c* Trimmed muscle fragments from SM and LD muscle of 4- to 5-d-old piglets were digested with 0.25% trypsin alone or with a combination of 0.25% trypsin with 0.2% collagenase and 0.01% DNase and enriched with Percoll density gradient centrifugation. (*a*) Cell yield, viability, and cell size of isolated cells were determined directly after isolation with the countess automated cell counter (Invitrogen, Karlsruhe, Germany) with integrated trypan blue staining. Mean cell yield is illustrated as *bars*, and individual values are given by *circles*. Viability and cell size are represented as means ± SD, *n* = 5 piglets. Digestion with TCD liberated more SC/MPC than digestion with trypsin alone, but the differences between treatments showed only a statistical trend (*p* = 0.096). Cell viability and cell size were similar for both digestion procedures. For the statistical analysis, a paired *t* test was performed, and *p* ≤ 0.05 was considered statistically significant. (*b, *c*) Cells were immunostained for Desmin and were analyzed with flow cytometry (*b*) or microscopically (cell nuclei were stained with DAPI) (*c*). Flow cytometric analyses were performed at various time points between day 3 and day 11 of culture. For both digestion procedures single values from at least five different isolations and the resulting trend lines (T) are presented. The immunofluorescence stainings were conducted at day 7 of culture and results are representative of three individual experiments from three animals. Scale bar represents 50 μm. Desmin expression increased during culture and reached about 60% at days 6–8 in the two differently liberated SC/MPC cultures.

**Figure 2:**
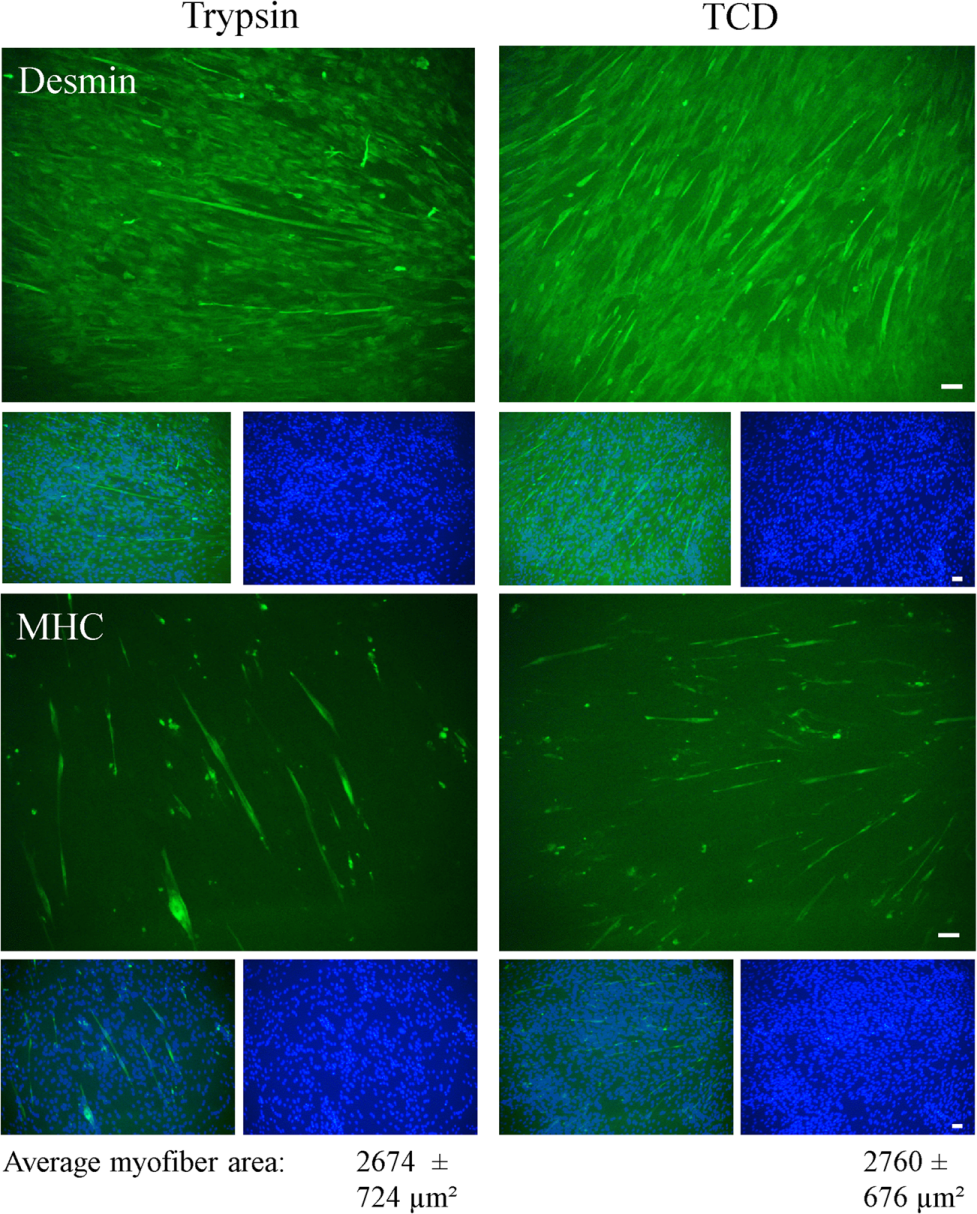
Differentiation potential of SC/MPC liberated with trypsin alone or with TCD. Isolated cell populations were seeded on Matrigel-coated plates at days 3–6 after isolation, and first cultured in growth medium. Subconfluent cells (about 80%) were transferred to differentiation medium after 4 to 5 d to induce myogenic differentiation. After 5 d in differentiation medium, cells were fixed and immunostained for Desmin and MHC to indicate their differentiation potential; cell nuclei were stained with DAPI. Quantitative analysis of differentiation was performed by encircling MHC+ myotubes (≥ 2 nuclei) to determine myotube area and results are illustrated in the figure as mean ± SD. SC/MPC dissociated with trypsin and TCD were both able to initiate myogenic differentiation (MHC+ cells) and started to form multinucleated myotubes. The images are representative of three individual experiments (*n* = 3 piglets) and 5–6 ramdom sections were evaluated per experiment. *Scale bar* represents 50 μm.

In 1974, Bischoff found that trypsin, in contrast to collagenase, effectively digested components of the basal lamina and sarcolemma membrane in the muscle tissue (Bischoff 1974). This membrane decomposition led to an effective release of SC. In bovine muscles, trypsinization resulted in more liberated cells than did collagenase digestion (Lee et al. 2007). However, trypsin is known to be relatively ineffective in dissociating extracellular matrix proteins (Santangelo 2008). Thus, we tested a combined trypsin, collagenase, DNase digestion, as described by Ortenblad et al. (2003) in order to test whether a combined digestion procedure would lead to better tissue dissociation and, as a result, to higher cell yield and to changes in *in vitro* cell function. Synergistic effects of mixed enzyme protocols were described for the isolation of other cell types such as adipose stromal vascular cells (Lockhart et al. 2015). Additionally, DNase I was added to the enzymatic dissociation mixture to digest liberated nucleic acids. Because of cell damage, nucleic acids are released into the dissociation medium and cause increases in viscosity and recovery problems (Santangelo 2008). Muscles (SM, LD) from early postnatal piglets (4- to 5-d-old) were used in this study.

By trend, we liberated more cells with the combined digestion procedure compared with trypsin alone which also resulted in more cells during culture. The higher cell yield obtained by using the combined enzyme digestion procedure presumably relies on the dissociation of the connective tissue by collagenase. Skeletal muscle consists of approximately 80–90% muscle fibers and 10–20% connective and fat tissues (Alvarenga et al. 2013; Listrat et al. 2016). We consider that the combination of trypsin and collagenase digests the basement membrane and the underlying connective tissue more effectively than trypsin alone, resulting in more liberated cells. In comparison with adult pigs, the extracellular matrix organization (e.g., collagen fibril arrangement) of newborns is less dense and regular (Wojtysiak 2013), giving one possible explanation why the effect is not statistically significant. Second, the high variability can also be caused by high litter and animal variations in pigs (Pardo et al. 2013). Therefore, the maturity of the used animals should always be considered in the optimization of tissue digestion.

Other studies have shown that the various enzymes or enzyme cocktails used in tissue digestion can release different populations of cells (Allalunis-Turner and Siemann, 1986). In our experiments, the phenotype and the *in vitro* functions (cell size, viability, proliferation, Desmin expression, and myogenic differentiation) of the isolated cells did not differ between the tested digestion procedures. We consider that the addition of the collagenase and DNase to trypsin increased the efficiency of tissue dissociation but neither altered cell function nor released other cell types in our experimental setting. Since we only analyzed the enriched myogenic cells from the 40/70% Percoll layer, differences in cell function and the released cell types may become apparent without use of Percoll gradient density centrifugation.

In various studies (Ortenblad et al. 2003; Nissen et al. 2005; Theil et al. 2006 Nissen and Oksbjerg 2009), muscle tissue dissociation was conducted with collagenase type II, which contains higher levels of trypsin-like activity, mainly attributable to clostripain (Santangelo 2008; Stahle et al. 2015). Collagenase batches containing higher levels of tryptic activity are more efficient in pancreas islet isolation (Brandhorst et al. 2009). As trypsin was present in both digestion procedures in our study, we do not think that the lower protease activity of collagenase type I is an influencing factor in our experiments. In contrast to trypsin, collagenase is known to be severely suppressed in the absence of Ca^2+^ and might thus not exert its full activity in Ca^2+^-free HBSS solution. However, the higher cell yield obtained with the combinatorial treatment suggests normal collagenase activity. This can be explained by the release of Ca^2+^ from extracellular matrix components, destroyed cells, and cell debris during the tissue digestion procedure (Maurer and Hohenester 1997).

In conclusion, the present experiments demonstrate that trypsinization and a combined digestion procedure (trypsin with collagenase and DNase) are suitable for isolating viable, myogenic, and functional SC/MPC from the muscles of early postnatal piglets. In addition, SC/MPC *in vitro* functions were similar. Cell yields obtained with the combined digestion procedure tends to be higher but show a high variability that seems to reflect marked differences in the developmental state of the muscles of individual neonatal piglets. However, because connective tissue organization changes in older pigs, simple trypsinization might lead to an incomplete dissociation of the muscle tissue in adults. Therefore, the more combinatorial type of tissue digestion could be more helpful for SC/MPC isolation from muscles of juvenile and adult pigs.
